# Establishing selectivity of FAK-paxillin PPI inhibitor using pulldown proteomics and a focal adhesion protein selectivity panel

**DOI:** 10.1016/j.bbrep.2025.102410

**Published:** 2025-12-14

**Authors:** Hunter O'Brien, Brock Hay, Huzaifah Sheikh, Liam McCreary, Krishna Parsawar, Timothy Marlowe

**Affiliations:** a Department of Internal Medicine, University of Arizona College of Medicine - Phoenix, 475 N. 5th Street, Phoenix, AZ, 85004, USA; b University of Arizona Analytical & Biological Mass Spectrometry Facility, 1657 E. Helen Street, Tucson, AZ, 85721, USA

**Keywords:** Surface plasmon resonance, Interactome, Proteomics, Mass spectrometry, Drug selectivity, Peptide therapeutic

## Abstract

UA-2012 (and related non-myristoylated analog UA-1907) is a lead alpha-helical cyclic peptide which inhibits the focal adhesion kinase (FAK)-paxillin protein-protein interaction (PPI) and is being evaluated for the treatment of cutaneous melanoma. However, the development of an empirical approach to measure PPI inhibitor selectivity remains an important need. We report the development of a pulldown-MS proteomic approach, including a custom synthesized non-myristoylated UA-1907-agarose probe, to evaluate the binding selectivity of candidate FAK PPI inhibitors. Melanoma lysates were probed with UA-1907-conjugated agarose beads and eluted associated proteins were analyzed through untagged mass-spectroscopy proteomics. The identified proteins led to the development of a custom focal adhesion (FA) selectivity panel comprised of recombinant VinT, VinH, PARVA, PARVB, Talin-1 Rod 8, and the FAK FAT domain. Surface plasmon resonance (SPR) screening of these FA proteins against UA-1907 determined that only the FAK-FAT domain has a nanomolar binding affinity (K_D_) for UA-1907, whereas other FA proteins have no binding. Overall, we report the development of a customized pulldown-MS approach to characterize PPI drug selectivity that has utility in the FAK drug discovery field.

## Highlights

• FAK-FAT inhibitor UA-1907 (UA-2012 analog) binds with nanomolar affinity to the FAT domain of FAK.
• Development of a pulldown-MS approach to identify peptide drug binding interactors.
• UA-1907 shows specificity to FAK-FAT amongst a focal adhesion selectivity panel.

## 1 Introduction

Despite clinical improvements provided by checkpoint inhibitors and targeted therapeutics, cutaneous melanoma is still the most fatal skin cancer worldwide with a strong need for new targeted approaches [1]. Focal adhesion kinase (FAK) is a dual kinase and scaffolding protein that is upregulated in numerous solid-tumor cancers [2], including melanoma [3]. ATP-competitive inhibitors of the FAK kinase domain have been clinically evaluated for the treatment of several solid tumors; however, results have been largely unsuccessful as a monotherapy [4]. Due to high conservation of the FAK ATP-binding pocket, kinase inhibitor selectivity remains a prominent issue as well as endogenous transphosphorylation resistance mechanisms upon FAK kinase inhibition [5,6]. FAK kinase inhibitors have demonstrated poor selectivity in kinome arrays, including off-target inhibition of kinases JAK2/3, CDK1, CDK7, AURKA, AXL, etc. [5,7]. Alternative approaches to target FAK in solid tumors have been explored in recent years, including proteolysis-targeting chimeras (PROTACs) [8], which degrade FAK protein levels, as well as inhibitors of FAK protein-protein interactions (PPIs), specifically the inhibitors of the FAK-paxillin interaction [5,9]. In melanoma, inhibition the FAK-paxillin interaction has been shown to be an effective therapeutic strategy, as demonstrated in several reports using site-directed mutagenesis, dominant-negative constructs, and therapeutic peptides [5,10].

Paxillin is a highly dynamic focal adhesion (FA) adaptor protein that directly interacts with FAK to promote tumor cell survival, attachment, invasion, and metastasis [11,12]. Paxillin contains five LD domains, four LIM domains, and several tyrosine/serine phosphorylation sites directly responsible for signaling pathways related to FA maturation, turnover, and lamellipodia formation [12,13]. Paxillin is overexpressed in numerous cancers, including melanoma [12,14]. The interaction between Paxillin's LD2 and LD4 domains and the FAK focal adhesion targeting (FAT) domain is critical for the FAK localization to FAs and drives FA maturation [15,16]. Due to both FAK and paxillin upregulation in cancer, inhibition of the FAK-paxillin PPI is a promising therapeutic approach [12].

In an effort to inhibit the FAK-paxillin PPI, our group has recently designed and developed a cyclic peptide, UA-1907, and a cell-permeable myristoylated version, UA-2012, based on the alpha-helical paxillin LD2 sequence that binds the FAK FAT domain with high affinity [5]. In a previous report, we solved the X-ray co-crystal structure of the inhibitor with the FAK FAT domain, demonstrated its mechanism-of-action through delocalization of FAK from focal adhesions, and showed its anti-cancer effects on tumor cell survival and invasion using *in vitro* and *in vivo* model systems of melanoma [5]. Specifically, UA-2012 showed superiority to FAK-kinase inhibitors in target selectivity, cancer: normal cell selectivity, and induction of cancer cell apoptosis. Because UA-2012 is a peptide derived from a naturally occurring specific PPI and the FAK FAT domain is highly unique with low sequence conservation, we hypothesized that the drug selectivity of UA-2012 would be much greater than ATP-competitive FAK inhibitors. However, we have yet to fully evaluate the binding selectivity of UA-2012 using proteomic approaches and a comprehensive panel of related focal adhesion proteins.

Numerous experimental approaches exist to probe the kinome selectivity of ATP-competitive kinase inhibitors, including competitive bead-based inhibition assays (e.g., KINOMEscan [17]), chemical proteomics [18], and computational modeling [19]. However, a current limitation in the PPI drug discovery field is a universally accepted experimental technique to probe the selectivity of candidate PPI inhibitors. In this report, we describe the development of a pulldown-mass spectrometry (MS) approach to evaluate the binding and selectivity of non-myristoylated FAK PPI inhibitor, UA-1907. We synthesize an analog of UA-1907 that was covalently coupled to NHS-agarose beads for pulldown experiments and validated its functionality using FAK FAT protein. Utilizing 1907-agarose as a chemoproteomic tool, we perform pulldown assays from melanoma cell lysate followed by LC/MS-MS proteomic analysis. Top protein hits are evaluated by gene ontology and STRING protein-protein interaction analysis, which revealed FAK, as well as network of focal adhesion proteins, as the top candidates forming a complex with UA-1907. To evaluate direct vs. indirect binding to UA-1907, we produce a panel of six recombinant focal adhesion proteins and tested binding via SPR, confirming direct binding only between FAK and UA-1907. In summary, we report the development of a customized pulldown-MS approach to characterize PPI drug selectivity that has utility in the FAK drug discovery field.

## 2 Materials and methods

### 2.1 Peptide synthesis, purification, and characterization

UA-1907 R7K peptide was synthesized using solid-phase peptide synthesis methodology and the Biotage Initiator + Alstra automated microwave-assisted peptide synthesizer as previously described [5]. H-Rink Amide ChemMatrix resin was utilized for peptide synthesis. Peptide was purified on an Agilent 1260 II quaternary HPLC on Zorbax SB-C18 (Agilent 880975–202) column. Peptide characterization was performed on an Agilent 1200 HPLC on Zorbax SB-C18 (Agilent 830990–902) column. The chemical structure of UA-1907 R7K and LC-MS trace is found in Fig. S1, which confirms purity >99 %.

### 2.2 Peptide conjugation to agarose beads

UA-1907 and/or UA-2014 (inactive control, described in Ref. [5]) R7K peptide was covalently conjugated to NHS-Activated Agarose Dry Resin (Thermo Fisher, Cat. No. 26196) following the manufacturer's protocol. NHS-activated dry resin was hydrated with peptide solution in 100 mM TEAA pH 9 buffer. The coupling reaction was conducted at RT for 1 h. LCMS was used to monitor the reaction. Following conjugation, the resin was washed with PBS and quenched with 1 M ethanolamine. Resin was stored at 4 °C and protected from light until use in pulldown experiments.

### 2.3 Cell culture and lysate production

Melanoma cell line SK-MEL-147 was grown in DMEM media (5 % Penicillin and Streptomycin). Cell were lysed in NP-40 buffer from RPI (50 mM TRIS/TRIS HCL, 150 mM NaCl, 1 % NP-40, 5 mM EDTA, pH 7.2–7.6), 10 % glycerol, 1X protease and phosphatase (ThermoFisher) inhibitors. Lysate was agitated on ice, centrifuged at 13,000 RCF for 30 min, supernatant was extracted and ran in a BCA protein assay (ThermoFisher). Absorbance was read on the CLARIOstar Plus (BMG Labtech) plate reader. Concentrations were recorded and lysates were stored at −80C.

### 2.4 Pulldown-mass-spectrometry

3000ug of SK-MEL-147 lysates were precleared in 60uL agarose beads for 1 h at 4C. Precleared lysate was incubated with 120uL of UA-1907 beads (or inactive control UA-2014 beads) o/n, rotating at 4C. Beads were were washed with NP40 buffer without inhibitors and proteins were eluted using 63 mM Tris pH 6.8 and 5 % SDS. Elution proteins underwent trypsin digestion and were analyzed in nanoLC-MS/MS at the UA Biological Mass Spectroscopy Core using the Thermo Q Exactive Orbitrap mass spectrometer (MS) coupled to the Thermo Vanquish Neo nano-LC system. Peptide sequences were determined, correlated to SwissProt protein sequences, and normalized in Scaffold Proteomics Discoverer.

### 2.5 Gene ontology and STRING analysis

Protein Label Free Quantification (LFQ) was represented by Normalized Total Spectra. Fold Enrichment (FE) was calculated using Eq. (1):

$$FE=\frac{\left(Pulldown\;LFQ-Ethanolamine\;Control\;Bead\;LFQ\right)}{Total\;Lysate\;LFQ}$$

Proteins were sorted by Fold Enrichment value to identify the top 100 proteins and further sorted by LFQ >20 to limit overcorrection bias. The filtered list of 40 proteins were ran on the NIH DAVID bioinformatics tool (DAVID Knowledgebase v2025_1) [20]. Gene Ontology categories for GOTERMS BP_DIRECT, CC_DIRECT, and MF_DIRECT were selected. The top 5 GO terms by p-value were selected. The top 40 proteins were analyzed for protein-protein association network by STRING (Version 12.0) [21].

### 2.6 Western blotting

Bead elutions were ran on a 4–20 % TGX Stain Free Gel and imaged via Stain Free Imaging (ChemDoc Biorad Imager) before transfer onto a PVDF membrane (TurboTransfer High MW). Blots were blocked using 5 % BSA in TBS-0.1 % Tween-20. Each primary antibody was used in 1:1000 dilution and incubated overnight at 4C. Blots were washed 3X and incubated in 1:5000 secondary antibody at RT for 1 h. Blots were incubated with ECL reagent (Biorad) for 5 min. Blots were imaged utilizing ChemDoc Biorad Imager for 3 min taking 60 total images. Results can be found in Supplementary Fig. 2.

### 2.7 Protein expression and purification

Vinculin Tail (VinT) (a.a. 811–1066), Vinculin Head (VinH) (a.a. 1–258), Actopaxin, (PARVA complete protein), beta-parvin (PARVB, total protein), FAK FAT (a.a. 892–1052), and Talin-1 Rod 8 (a.a. 1453–1580) were purified in *E. coli* BL21 DE3 using 6X-Histag TOPO-100 plasmid constructs with AmpR gene (ThermoFisher). Purification procedures were as previously described [5]. Proteins were purified using NiNTA affinity chromatography (ThermoFisher) followed by size-exclusion chromatography using the Akta Pump 900 system and HiLoad 26/600 SuperDex 75 pg column. Major peaks (Unicorn 3.0 software) were validated using SDS-PAGE stain free imaging (Biorad).

#### 2.7.1 Surface plasmon resonance (SPR)

SPR was performed on a Sartorius Pioneer FE instrument as previously described [5]. Briefly, biotinylated UA-1907 was immobilized onto Streptavidin Dextran Hydrogel (SADH) biosensor at a RU = 50. Proteins were flowed over at 75 μL/min in running buffer (50 mM Tris pH 8.0, 200 mM NaCl, 5 % glycerol) from 2.5 μM to 4.9 nM. Equilibrium dissociation constant (K_D_) was calculated using Qdat software and 1:1 kinetic binding model.

## 3 Results

### 3.1 UA-1907 agarose bead conjugation and validation of activity

**Fig. 1:**
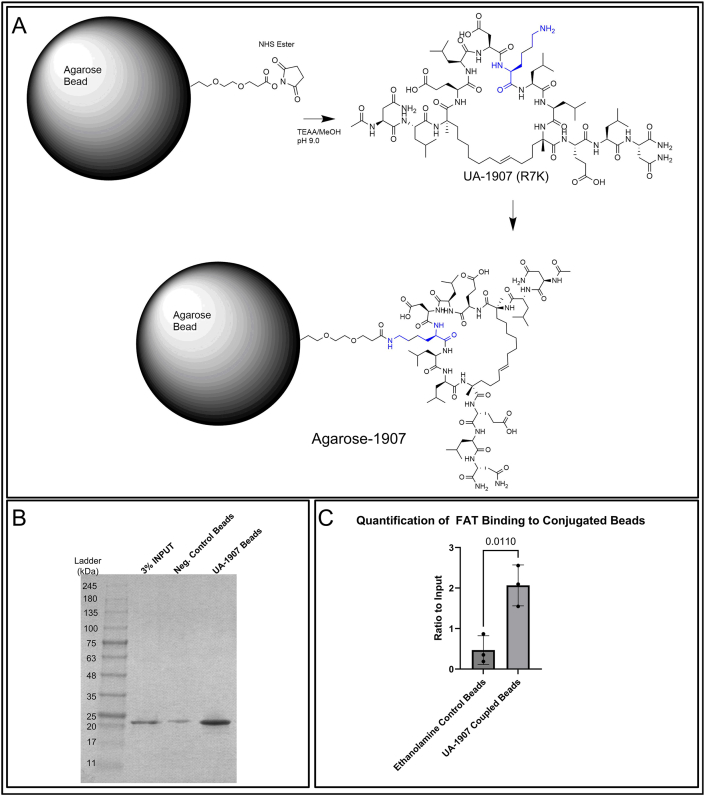
**Conjugation of UA-1907 peptide to agarose beads and characterization of FAT binding activity. A.** Schematic design of UA-1907 R7K conjugation to NHS-ester agarose via amine coupling. **B.** Coomassie gel image of UA-1907 pulldown using purified FAK FAT protein, which is known to bind to UA-1907. C. Quantification of UA-1907/FAT pulldown utilizing densitometry. Bands were measured by integrated density and compared to input. Unpaired *t*-test performed, p-value = 0.011. N = 3 independent biological replicates.

To create a tool to understand the binding interactions of UA-1907, we covalently cross-linked UA-1907 to agarose beads (Fig. 1a) using standard amine coupling chemistry. We utilized activated N-Hydroxysuccinimide (NHS)-ester modified agarose beads and synthesized a R7K derivative of UA-1907 (Fig. S1) to introduce a primary amine. Position 7 of the peptide was selected as the coupling site, as a previous report [5] detailed tolerability of this site for modification (e.g., TAMRA) without altering peptide activity. Successful crosslinking of peptide to agarose beads was confirmed by LC-MS. Beads were then analyzed for activity using positive control FAK FAT protein as a quality control test. As shown in Fig. 1b, UA-1907-beads enriched binding to FAK FAT, significantly more than negative control beads (ethanolamine). Quantification of UA-1907 pull-down results (Fig. 1c) showed a 4-fold increase in FAK FAT binding compared to neg. control beads (p = 0.011). Overall, we successfully coupled UA-1907 to agarose beads and validated their activity in pull-down binding assays to FAK FAT, qualifying use in LC/MS-MS proteomic studies.

### 3.2 LC/MS-MS pulldown results of UA-1907 in melanoma lysate

**Fig. 2:**
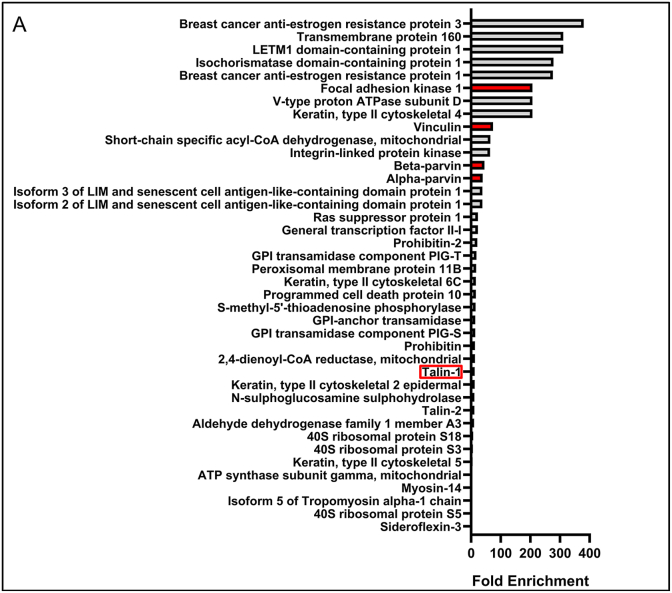
**Identification of UA-1907 associated proteins by SKMEL-147 lysate pulldown-LC-MS/MS.** UA-1907 pulldown assay performed in SKMEL-147 melanoma cell lysate. Proteins were eluted and analyzed via LC-MS/MS. Proteins identified by LC-MS/MS sorted by fold enrichment and label free quantification >20. Proteins identified for future purification and binding analysis are colored in red. Proteins selected based on label free quantification, fold enrichment, and previous evidence demonstrating paxillin binding.

To evaluate the binding complex that UA-1907 forms in melanoma cells, we performed pulldown assays using UA-1907- and inactive control peptide (UA-2014)-agarose beads incubated with SK-MEL-147 melanoma cell lysate and processed samples for LC/MS-MS proteomic analysis. Ethanolamine treated beads additionally accounted for non-specific binding and lysate input samples were used to calculate fold enrichment of binding. After LC/MS-MS analysis, protein/peptide quantification was assessed with Label Free Quantification (LFQ) and Fold Enrichment calculation (equation (1)). As common with proteomic analysis, pulldown-MS of SKMEL-147 melanoma cell lysate with UA-1907 agarose beads identified >1000 unique peptide sequences. SKMEL-147 was selected as the experimental model due to previous findings showing significant efficacy after treatment with FAK-paxillin inhibitor UA-2012 [5]. To filter out bona fide vs. background interactions, protein hits were ranked by fold enrichment (Fig. 2 & Table S1). Top hits included known FA proteins focal adhesion kinase 1, vinculin, talin-1, alpha-parvin, beta-parvin, and BCAR1 (p130Cas). Intriguingly, these FA proteins have an established role as part of the FA complex and paxillin binding [[22], [23], [24], [25]]. Of note, we saw specificity in these top hits, showing no fold enrichment after pulldown with our inactive peptide control UA-2014 (Table S1). Additional hits were identified, including BCAR3, LETM1 domain-containing protein 1, and transmembrane protein 160. Overall, the UA-1907 pulldown-MS experiment successfully enriched for multiple members of the focal adhesion complex.

### 3.3 Proteomic gene ontology and protein-protein interaction network analysis

**Fig. 3:**
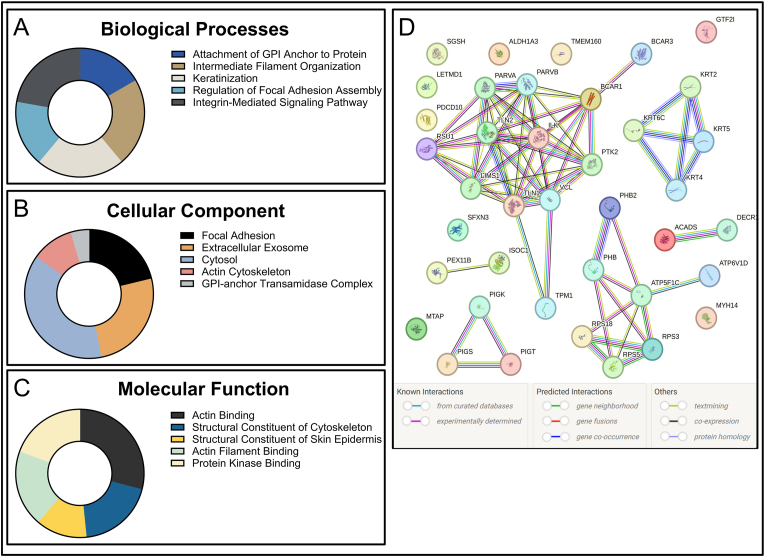
**Gene Ontology and STRING analysis of UA-1907 pulldown results.** Gene Ontology for the most enriched proteins detected with respect to **A.** Biological Processes, **B.** Cellular Component (Location), and **C.** Molecular Function. GO Terms and enrichment scores were generated using the NIH DAVID bioinformatics service. Pathways were sorted by the lowest p-value (highest enrichment) and the top 5 were represented per group. The values in the pie charts correspond to the number of genes present in each enriched pathway. Expanded GO terms are available in Table S2–4. **D.** STRING analysis for the most highly enriched protein-protein interaction networks detected in UA-1907 pulldown. Edge lines represent predicted and known PPIs by their source: curated databases (cyan), experimentally (pink), gene neighborhood (green), gene fusions (red), gene co-occurrence (blue), textmining (yellow), co-expression (black), and protein homology (purple).

Utilizing NIH's DAVID software, we performed gene ontology analysis on the UA-1907 pulldown results [20]. As expected, the most enriched biological processes (Fig. 3a, Table S2) included integrin-mediated signaling pathway, regulation of focal adhesion assembly, and intermediate filament organization. The cellular component analysis (Fig. 3b–Table S3) followed a similar trend, with enriched pathways being focal adhesion, actin cytoskeleton, and cytosol. Intriguingly, we saw association with GPI anchor complexes in both BP and CC ontologies, which may indicate FAK association with these cell membrane anchorage structures. Molecular function (MF) (Fig. 3c–Table S4) showed enrichment in actin binding, structural constituent of cytoskeleton, and protein kinase binding, further supporting the focal adhesion complex as the primary target of UA-1907.

We next performed STRING analysis (Fig. 3d) to map the protein-protein complex pulled down by UA-1907 [21]. STRING analysis found a strong association between known UA-1907 target PTK2 (FAK) and other focal adhesion proteins talin-1 (TLN-1), vinculin (VCL), alpha-parvin (PARVA), beta-parvin (PARVB), integrin-linked kinase (ILK), LIMS1 (PINCH-1), and BCAR1 (p130cas). Notably, the FAK interaction network did not contain paxillin (PXN), presumably due to inhibition by competitive inhibitor UA-1907. Additional protein-protein interaction networks included a keratin network, mitochondrial protein synthesis, and GPI transamidase network. Overall, these STRING analyses confirmed a FAK-containing interactome as a major target of peptide UA-1907.

### 3.4 Pulldown-WB confirms UA-1907 enriches focal adhesion proteins

To confirm that UA-1907 pulldown enriches for focal adhesion proteins using an orthogonal method, we performed pulldown-WB experiments. Proteins FAK, talin, vinculin, and paxillin were evaluated. WB analysis demonstrated that UA-1907 beads selectively enriched the focal adhesion proteins FAK, vinculin, and talin (Fig. S2a). In contrast, paxillin was not enriched, consistent with the compound's role as a paxillin-competitive inhibitor. Negative control beads showed insignificant binding, confirming the specificity of these interactions. Densitometry analysis further supported these findings (Fig. S2b), revealing strong enrichment of FAK (p-value = 0.0027), vinculin (p-value = 0.0096), and talin (p-value = 0.0041), whereas paxillin showed no significant enrichment (NS). Together, these findings indicate that UA-1907 forms a complex with focal adhesion proteins FAK, talin, and vinculin, while excluding paxillin from the complex.

### 3.5 Purification of recombinant focal adhesion protein panel

Because the FA complex contains over 60 different proteins, it is plausible that interactions detected in the UA-1907 pull-down assay may be indirect. To help filter out direct vs. indirect UA-1907 interactions, we expressed and purified 6 distinct FA proteins (FAK FAT domain, TLR8, PARVA, PARVB, VinH, and VinT) using Ni-NTA affinity chromatography. These FA proteins were selected based on pulldown-MS results, as well as previous evidence demonstrating binding to LD motifs [22,24]. Following affinity chromatography, our 6-protein focal adhesion panel was subsequently purified by size exclusion chromatography and MW were confirmed by SDS-PAGE and Coomassie staining (Fig. S3a–f).

### 3.6 Confirmation of UA-1907 direct binding to protein targets via SPR

**Fig. 4:**
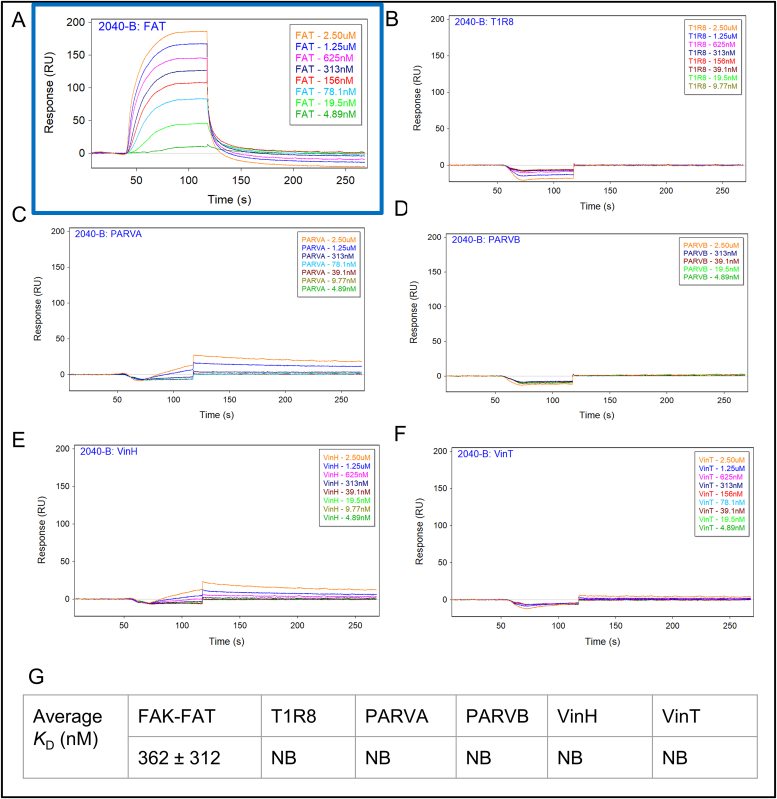
**FA Selectivity Panel SPR experiments.** Biotin-conjugated UA-1907 (2040) was immobilized onto SADH biosensors at a RU of 50 on the Sartorius Pioneer FE instrument. Purified focal adhesion proteins were injected through the 2040 channel at 75uL/min in a dose-response manner. SPR sensorgrams of distinct FA proteins are represented in **A.** FAT, **B.** T1R8, **C.** VinH, **D.** VinT, **E.** PARVA, and **F.** PARVB. **G.** The average K_D_ calculated from all replicates (n = 3) performed based on kinetic analysis available on QDat software. Concentrations: 2.5 μM - 4.9 nM. NB: No Binding detected.

To evaluate the direct binding affinity of UA-1907 to our 6-protein focal adhesion panel, we ran our purified recombinant proteins on SPR. Biotinylated UA-1907 was immobilized onto a SADH biosensor and FA proteins were flowed over from 4.9 nM to 2.5 μM. FAK FAT (Fig. 4a) bound to UA-1907 in a dose-dependent manner, with a *K*_D_ of 362 nM (Fig. 4a and g). Conversely, TLR8 (Fig. 4b), PARVA (Fig. 4c), PARVB (Fig. 4d), VinH (Fig. 4e), and VinT (Fig. 4f) and showed minimal binding to UA-1907, even at the max 2.5 μM concentration. Calculated *K*_D_ values of UA-1907 binding to TLR8, PARVA, PARVB, VinH, and VinT were all NB (no binding). Overall, the FAK FAT nK_D_ of 362 nM to UA-1907, with minimal binding to other focal adhesion proteins, supports the specificity of UA-1907. These data also suggest that UA-1907 pulldown may result in the isolation of the entire macromolecular focal adhesion complex, including both direct (e.g., FAK) and indirect (e.g., talin-1, vinculin, etc.) interactions.

## 4 Discussion

Since its discovery over 30 years ago [2], FAK has been identified as an attractive drug target in numerous cancers, including melanoma. Many FAK therapeutics have been developed, primarily targeting its kinase domain, with limited success as a monotherapy [26] due off-target kinase activity [7], rapid kinome reprogramming upon kinase inhibition [6], and the lack of effect on the major scaffolding function of FAK [5]. The FAT domain, responsible for mediation of multiple essential oncogenic pathways through its scaffolding domain, offers an attractive target for highly specific and effective anti-FAK therapy. Novel FAK candidate UA-1907 provides a solution to target FAK scaffolding function and focal adhesion localization as a cancer therapy.

LC-MS/MS in tandem with pulldown provides an innovative tool to identify protein binding to target molecules (e.g., UA-1907) in cellular lysate. Our findings from the pulldown identified multiple focal adhesion proteins, including FAK, talin-1, vinculin, alpha-parvin, beta-parvin, integrin-linked kinase, PINCH-1, p130cas (BCAR1), and BCAR3. Because FAK forms a large macromolecular focal adhesion complex with these proteins [27], it is likely that the identified secondary focal adhesion proteins in the UA-1907 pulldown assay represent indirect binding as partners in the focal adhesion complex rather than direct binding to UA-1907 itself. For example, FAK has been also shown to bind to talin-1 [28], which contains 11 vinculin binding sites [29], explaining the UA-1907 pulldown-WB results (Fig. S2). The SPR direct binding results corroborate these conclusions, showing direct binding of UA-1907 to only the FAK FAT domain. Key focal adhesion scaffolding proteins p130cas and BCAR3, which have a role in integrin and FAK signaling [30], may be investigated in future studies for complex formation with UA-1907. Intriguingly, paxillin did not pulldown with UA-1907, in either MS or WB experiments, indicating that UA-1907 disrupts FAK-paxillin interactions as part of its mechanism of action (MOA). These findings are consistent with our previous report [5], in which we demonstrated that UA-1907 dose-dependently inhibits the FAK-paxillin interaction in both cellular and biochemical systems.

Gene ontology and STRING analysis allowed for a functional outlook of the proteins identified via LC-MS/MS. A few common themes were seen throughout all GO and STRING analyses. Primarily, the most enriched pathways involved the focal adhesion, actin, and the cytoskeleton. The regulation of the actin cytoskeleton via FAK is a well reported finding amongst focal adhesion research [31]. Another enrichment was GPI-anchor complexes; while these complexes are not as directly related to pathways above, recent evidence suggests integrin beta 1 activation of FAK leads to downstream formation of GPI-AP nanoclusters [32]. Particularly, enriched focal adhesion proteins talin-1 and vinculin contribute to GPI anchor prominence. From all GO and STRING analyses, we can conclude that the focal adhesion complex is heavily enriched after binding to UA-1907.

The most significant finding in this manuscript is the nanomolar, highly specific affinity (Fig. 4g) between UA-1907 and the FAK FAT domain by SPR, but not TLR8, PARVA, PARVB, VinH, and VinT. As previously discussed, the challenge of targeting FAK historically has been the off-target effects of ATP-competitive kinase inhibitors [5]. For example, clinical-stage FAK kinase inhibitor, defactinib, has demonstrated poor kinome selectivity, making it difficult to characterize its FAK-specific pharmacological effects and causing high cellular toxicity [5]. The high selectivity of the FAT domain targeting allows for UA-1907 and myristoylated analog UA-2012 to be a viable candidate for not only melanoma but other FAK-mediated diseases (pulmonary fibrosis, cirrhosis, pancreatic cancer, etc.). Alongside its implications across many human cancers, FAK plays a prevalent role in both myofibroblast activation and progression of fibrotic disease [33]. UA-1907's high specificity and affinity may enable it to have strong therapeutic potential with limited off-target side effects.

In summary, we characterized the binding selectivity of novel FAK FAT inhibitor UA-1907, the non-myristoylated analog of UA-2012. Utilizing agarose beads conjugated to peptide, we identified key protein complexes bound to the drug via LC-MS/MS. We performed gene ontology and STRING analyses to better understand the interactome of binding partners to UA-1907. Using this information, we purified a panel of 6 recombinant focal adhesion proteins, which were subsequently utilized in SPR to identify their binding to UA-1907. Ultimately, we found that UA-1907 had strong nanomolar binding affinity to the FAT domain of FAK, while minimal affinity to other FA proteins. Overall, the specificity and strength of UA-1907 binding contributes to its potential as a highly specific FAK inhibitor for the treatment of melanoma and other diseases.

## CRediT authorship contribution statement

**Hunter O'Brien:** Conceptualization, Data curation, Formal analysis, Investigation, Methodology, Software, Supervision, Validation, Visualization, Writing – original draft, Writing – review & editing. **Brock Hay:** Conceptualization, Data curation, Formal analysis, Investigation, Methodology, Software, Validation, Visualization, Writing – original draft. **Huzaifah Sheikh:** Conceptualization, Formal analysis, Investigation, Software, Visualization, Writing – review & editing. **Liam McCreary:** Conceptualization, Investigation, Methodology, Writing – original draft. **Krishna Parsawar:** Conceptualization, Investigation, Methodology, Software, Writing – review & editing. **Timothy Marlowe:** Conceptualization, Data curation, Formal analysis, Funding acquisition, Investigation, Methodology, Project administration, Resources, Software, Supervision, Validation, Visualization, Writing – original draft, Writing – review & editing.

## Declaration of competing interest

The authors declare the following financial interests/personal relationships which may be considered as potential competing interests: Timothy Marlowe reports a relationship with FAKnostics, LLC that includes: board membership, employment, and equity or stocks. If there are other authors, they declare that they have no known competing financial interests or personal relationships that could have appeared to influence the work reported in this paper.

## Data Availability

All data generated or analyzed during this study are included in this article.

## References

[1] Beaumont K.A., Mohana-Kumaran N., Haass N.K. (2013). Modeling melanoma in vitro and in vivo. Healthcare (Basel).

[2] Weiner T.M. (1993). Expression of focal adhesion kinase gene and invasive cancer. Lancet.

[3] Hess A.R., Hendrix M.J. (2006). Focal adhesion kinase signaling and the aggressive melanoma phenotype. Cell Cycle.

[4] Cance W. (2013). Disrupting the scaffold to improve focal adhesion kinase-targeted cancer therapeutics. Sci. Signal..

[5] Reyes L. (2025). Structure-based discovery of hydrocarbon-stapled paxillin peptides that block FAK scaffolding in cancer. Nat. Commun..

[6] Marlowe T.A. (2016). Oncogenic receptor tyrosine kinases directly phosphorylate focal adhesion kinase (FAK) as a resistance mechanism to FAK-kinase inhibitors. Mol. Cancer Therapeut..

[7] Slack-Davis J.K. (2007). Cellular characterization of a novel focal adhesion kinase inhibitor. J. Biol. Chem..

[8] Cromm P.M. (2018). Addressing kinase-independent functions of fak via PROTAC-mediated degradation. J. Am. Chem. Soc..

[9] Aromokeye R. (2025). Development of a high-throughput TR-FRET assay to identify inhibitors of the FAK-paxillin protein-protein interaction. SLAS Discov.

[10] Mousson A. (2021). Inhibiting FAK-paxillin interaction reduces migration and invadopodia-mediated matrix degradation in metastatic melanoma cells. Cancers (Basel).

[11] Antoniades I. (2021). FAK displacement from focal adhesions: a promising strategy to target processes implicated in cancer progression and metastasis. Cell Commun. Signal..

[12] Liu W. (2023). The role of paxillin aberrant expression in cancer and its potential as a target for cancer therapy. Int. J. Mol. Sci..

[13] Lopez-Colome A.M. (2017). Paxillin: a crossroad in pathological cell migration. J. Hematol. Oncol..

[14] Velasco-Velázquez M.A. (2008). Reduced paxillin expression contributes to the antimetastatic effect of 4-hydroxycoumarin on B16-F10 melanoma cells. Cancer Cell Int..

[15] Deramaudt T.B. (2014). Altering FAK-paxillin interactions reduces adhesion, migration and invasion processes. PLoS One.

[16] Antoniades I. (2017). Addressing the functional determinants of FAK during ciliogenesis in multiciliated cells. J. Biol. Chem..

[17] Karaman M.W. (2008). A quantitative analysis of kinase inhibitor selectivity. Nat. Biotechnol..

[18] Reinecke M. (2024). Chemical proteomics reveals the target landscape of 1,000 kinase inhibitors. Nat. Chem. Biol..

[19] Lo Y.C. (2019). Computational analysis of kinase inhibitor selectivity using structural knowledge. Bioinformatics.

[20] Huang da W., Sherman B.T., Lempicki R.A. (2009). Systematic and integrative analysis of large gene lists using DAVID bioinformatics resources. Nat. Protoc..

[21] Szklarczyk D. (2023). The STRING database in 2023: protein-protein association networks and functional enrichment analyses for any sequenced genome of interest. Nucleic Acids Res..

[22] Zacharchenko T. (2016). LD motif recognition by talin: structure of the Talin-DLC1 complex. Structure.

[23] Lorenz S. (2008). Structural analysis of the interactions between paxillin LD motifs and alpha-parvin. Structure.

[24] Stiegler A.L. (2012). Structural basis for paxillin binding and focal adhesion targeting of beta-parvin. J. Biol. Chem..

[25] Palmer S.M. (2009). Lipid binding to the tail domain of vinculin: specificity and the role of the N and C termini. J. Biol. Chem..

[26] Infante J.R. (2012). Safety, pharmacokinetic, and pharmacodynamic phase I dose-escalation trial of PF-00562271, an inhibitor of focal adhesion kinase, in advanced solid tumors. J. Clin. Oncol..

[27] Horton E.R. (2016). The integrin adhesome network at a glance. J. Cell Sci..

[28] Chen H.C. (1995). Interaction of focal adhesion kinase with cytoskeletal protein talin. J. Biol. Chem..

[29] Gingras A.R. (2005). Mapping and consensus sequence identification for multiple vinculin binding sites within the talin rod. J. Biol. Chem..

[30] Cary L.A. (1998). Identification of p130Cas as a mediator of focal adhesion kinase- promoted cell migration. J. Cell Biol..

[31] Fabry B. (2011). Focal adhesion kinase stabilizes the cytoskeleton. Biophys. J..

[32] Kalappurakkal J.M. (2019). Integrin mechano-chemical signaling generates plasma membrane nanodomains that promote cell spreading. Cell.

[33] Ding Q. (2013). FAK-related nonkinase is a multifunctional negative regulator of pulmonary fibrosis. Am. J. Pathol..

