# Supplementary material for: Establishing selectivity of FAK-paxillin PPI inhibitor using pulldown proteomics and a focal adhesion protein selectivity panel
Source: Biochem Biophys Rep. 2025 Dec 14;45:102410. doi: 10.1016/j.bbrep.2025.102410 (PMC12765108; doi:10.1016/j.bbrep.2025.102410)
Supplement: Multimedia component 1 [file mmc1.docx]

**Supplementary Information-**

**O’Brien, H, et al. Establishing Drug Selectivity of FAK-PPI Inhibitors using Pulldown Proteomics and a Focal Adhesion Protein Selectivity Panel**

**Table of Contents:**

**Supplementary Figure 1.** LC-MS Analysis of UA-0001907 R7K peptide.

**Supplementary Figure 2.** Western blot analysis of proteins enriched by UA-1907 pulldown in SKMEL-147 lysates.

**Supplementary Figure 3:** Purifications of recombinant focal adhesion protein panel: FAK-FAT, T1R8, PARVA, PARVB ,VinH, and VinT.

**Supplementary Table 1.** Fold enrichment and label free quantification of top proteins in UA-1907/SKMEL-147 pulldown.

**Supplementary Table 2.** Gene Ontology Proteomic Enriched Pathways: Biological Processes.

**Supplementary Table 3.** Gene Ontology Proteomic Enriched Pathways: Cellular Component.

**Supplementary Table 4.** Gene Ontology Proteomic Enriched Pathways: Molecular Function.


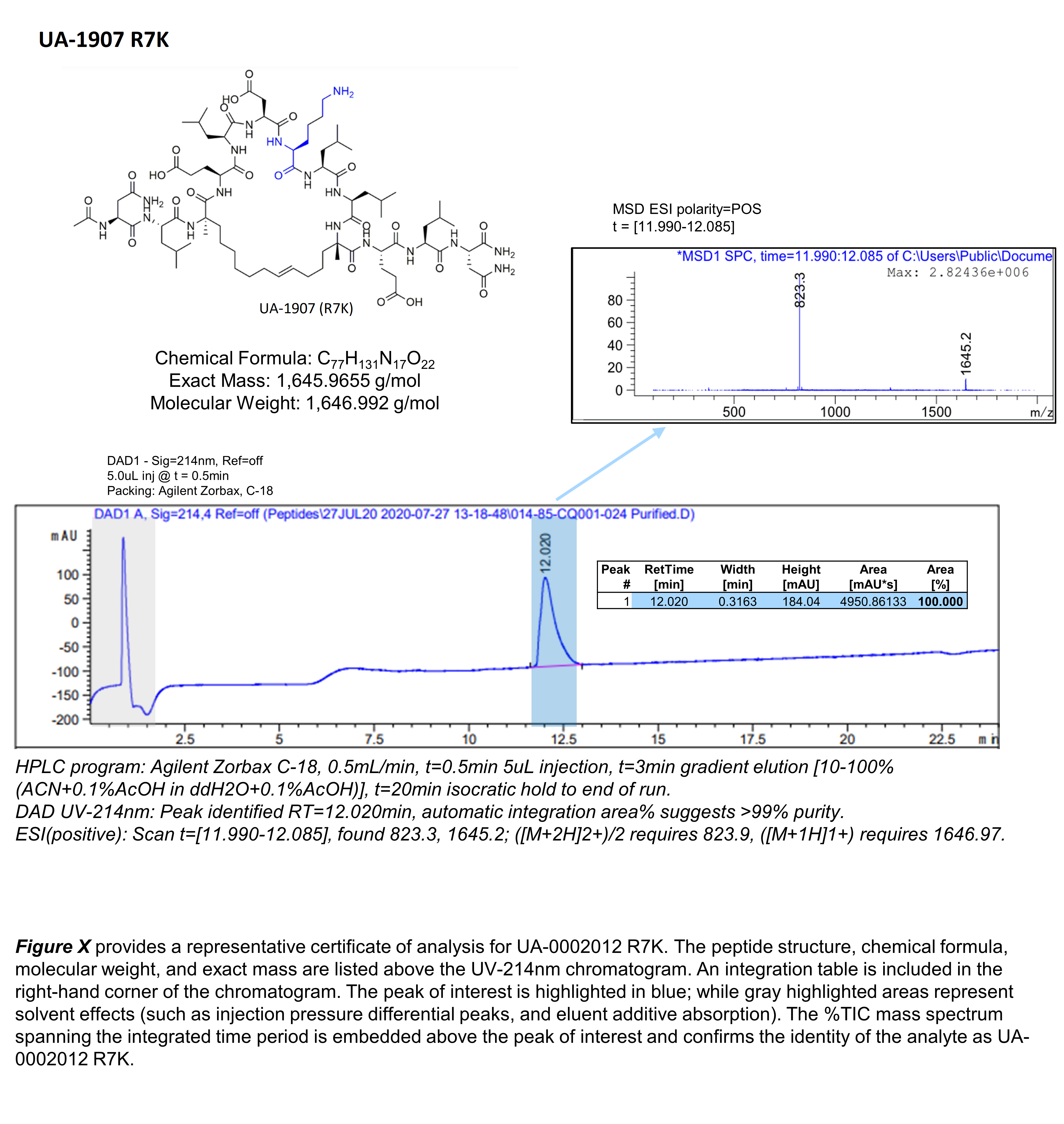


**Supplementary Figure 1. LC-MS Analysis for UA-0001907 R7K peptide.** The peptide structure, chemical formula, molecular weight, and exact mass are listed above the UV-214nm chromatogram. An integration table is included in the right-hand corner of the chromatogram. The peak of interest is highlighted in blue; while gray highlighted areas represent solvent effects (such as injection pressure differential peaks, and eluent additive absorption). The %TIC mass spectrum spanning the integrated time period is embedded above the peak of interest and confirms the identity of the analyte as UA-0001907 R7K.


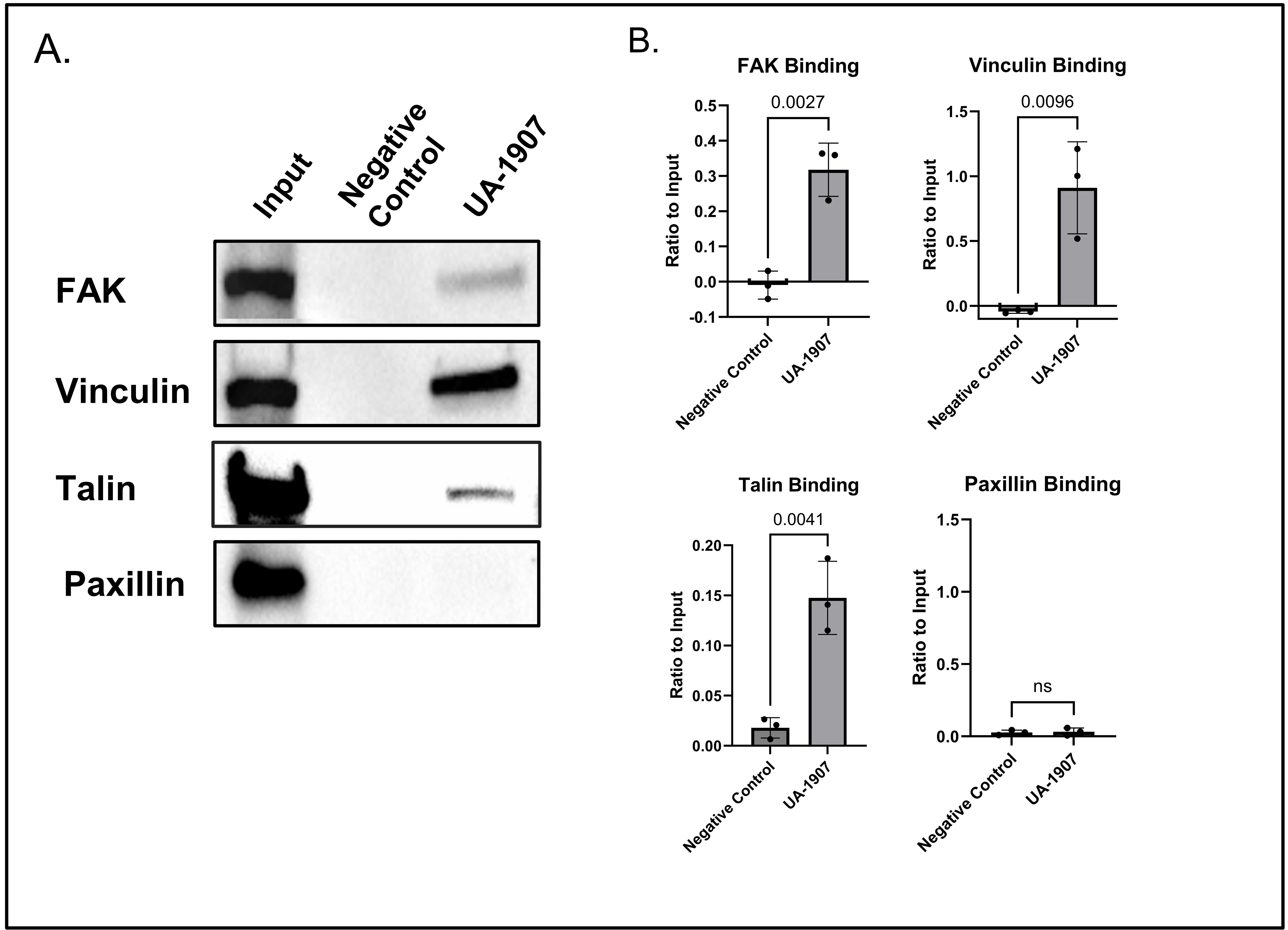


**Supplementary Figure 2. Western blot analysis of proteins enriched by UA-1907 pulldown in SKMEL-147 lysates.** (A) Western blot after UA-1907 pulldown experiment in SKMEL-147 lysates showing 3% input, ethanolamine-treated negative control beads, and UA-1907–conjugated agarose beads. Blots were probed for FAK, Vinculin, Talin, and Paxillin. Input represents 3% of the total lysate prior to pulldown. Ethanolamine beads served as a negative control for nonspecific binding. UA-1907–conjugated beads identified enriched proteins associated with the focal adhesion complex. (B) Quantification of pulldown enrichment by densitometry. Band intensities for each target protein were measured by integrated density and normalized to the 3% input. Compared with ethanolamine controls, UA-1907 beads showed a marked increase in binding of FAK, Vinculin, and Talin, indicating interaction of these adhesion-associated proteins with UA-1907. Unpaired t test performed, p-values indicated. N = 3 independent biological replicates.


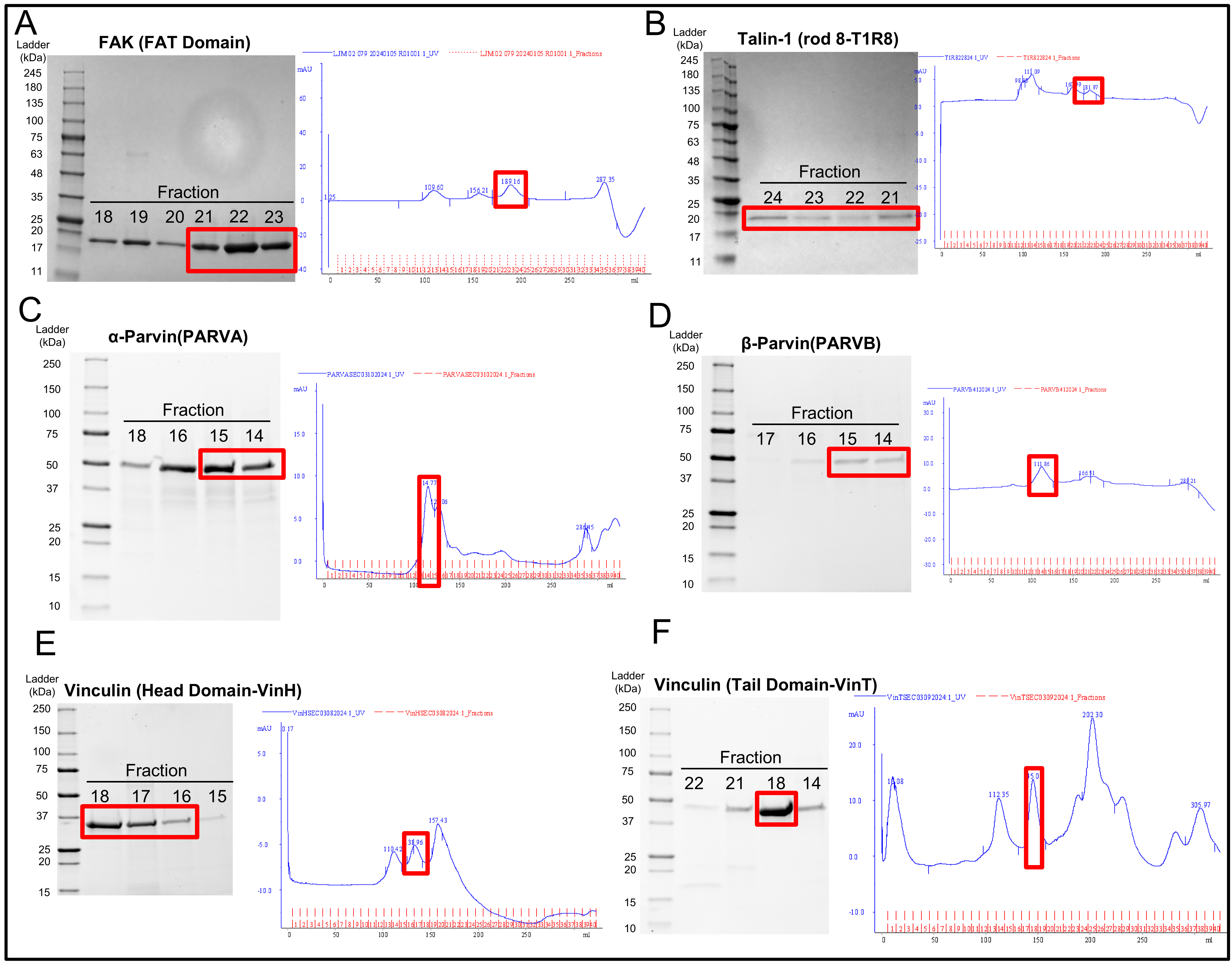


**Supplementary Figure 3: Purifications of recombinant focal adhesion protein panel: FAK-FAT, T1R8, PARVA, PARVB ,VinH, and VinT.** Constructs were designed with an N-terminal 6X His tag for affinity purification from BL21 *E. coli*. Proteins were subsequently purified by size-exclusion chromatography (SEC) and eluted fractions were ran on SDS-PAGE followed by Coomassie staining for **A.** FAK FAT, **B.** Talin-1 rod 8, **C.** PARVA, **D.** PARVB, **E.** Vinculin head (VinH), and **F.** Vinculin tail (VinT). Fractions identified in red boxes correspond to the correct molecular weight as observed by SEC and SDS-PAGE. Selected peaks were combined and quantified for running on SPR.

| **Identified Proteins** | **Accession Number** | **MW** | **1907 PQV** | **2014 (NC) PQV** | **1907 FE** | **2014 (NC) FE** |
| --- | --- | --- | --- | --- | --- | --- |
| Breast cancer anti-estrogen resistance protein 3 | O75815 | 93 kDa | 38 | 0 | 380 | 0 |
| LETM1 domain-containing protein 1 | Q6P1Q0 | 42 kDa | 31 | 9 | 311 | 88 |
| Transmembrane protein 160 | Q9NX00 | 20 kDa | 31 | 0 | 311 | 0 |
| Isochorismatase domain-containing protein 1 | Q96CN7 | 32 kDa | 31 | 0 | 278 | 0 |
| Breast cancer anti-estrogen resistance protein 1 | P56945 | 93 kDa | 28 | 0 | 276 | 0 |
| Keratin, type II cytoskeletal 4 | P19013 | 57 kDa | 21 | 0 | 207 | 0 |
| V-type proton ATPase subunit D | Q9Y5K8 | 28 kDa | 21 | 0 | 207 | 0 |
| **Focal adhesion kinase 1** | **Q05397** | **119 kDa** | **21** | **0** | **207** | **0** |
| **Vinculin** | **P18206** | **124 kDa** | **3,136.00** | **0** | **74** | **0** |
| Short-chain specific acyl-CoA dehydrogenase, mitochondrial | P16219 | 44 kDa | 31 | 0 | 65 | 0 |
| Integrin-linked protein kinase | Q13418 | 51 kDa | 370 | 0 | 64 | 0 |
| **Beta-parvin** | **Q9HBI1** | **42 kDa** | **86** | **0** | **45** | **0** |
| **Alpha-parvin** | **Q9NVD7** | **42 kDa** | **131** | **0** | **39** | **0** |
| Isoform 2 of LIM and senescent cell antigen-like-containing domain protein 1 | P48059 | 38 kDa | 166 | 0 | 38 | 0 |
| Isoform 3 of LIM and senescent cell antigen-like-containing domain protein 1 | P48059 | 44 kDa | 145 | 0 | 38 | 0 |
| General transcription factor II-I | P78347 | 112 kDa | 73 | 0 | 23 | 0 |
| Ras suppressor protein 1 | Q15404 | 32 kDa | 131 | 0 | 23 | 0 |
| Prohibitin-2 | Q99623 | 33 kDa | 190 | 4 | 21 | 0 |
| GPI transamidase component PIG-T | Q969N2 | 66 kDa | 35 | 0 | 18 | 0 |
| Peroxisomal membrane protein 11B | O96011 | 28 kDa | 28 | 0 | 17 | 0 |
| Programmed cell death protein 10 | Q9BUL8 | 25 kDa | 38 | 0 | 16 | 0 |
| Keratin, type II cytoskeletal 6C | P48668 | 60 kDa | 128 | 0 | 16 | 0 |
| S-methyl-5'-thioadenosine phosphorylase | Q13126 | 31 kDa | 83 | 0 | 15 | 0 |
| GPI transamidase component PIG-S | Q96S52 | 62 kDa | 21 | 0 | 14 | 0 |
| GPI-anchor transamidase | Q92643 | 45 kDa | 21 | 0 | 14 | 0 |
| 2,4-dienoyl-CoA reductase, mitochondrial | Q16698 | 36 kDa | 45 | 0 | 13 | 0 |
| Prohibitin | P35232 | 30 kDa | 145 | 2 | 13 | 0 |
| Keratin, type II cytoskeletal 2 epidermal | P35908 | 65 kDa | 463 | 0 | 12 | 0 |
| **Talin-1** | **Q9Y490** | **270 kDa** | **722** | **4** | **12** | **0** |
| Talin-2 | Q9Y4G6 | 272 kDa | 62 | 0 | 11 | 0 |
| N-sulphoglucosamine sulphohydrolase | P51688 | 57 kDa | 21 | 0 | 11 | 0 |
| Aldehyde dehydrogenase family 1 member A3 | P47895 | 56 kDa | 131 | 0 | 10 | 0 |
| 40S ribosomal protein S18 | P62269 | 18 kDa | 149 | 48 | 7 | 0 |
| 40S ribosomal protein S3 | P23396 | 27 kDa | 166 | 40 | 6 | 0 |
| Keratin, type II cytoskeletal 5 | P13647 | 62 kDa | 159 | 0 | 5 | 0 |
| Sideroflexin-3 | Q9BWM7 | 36 kDa | 24 | 2 | 4 | 0 |
| 40S ribosomal protein S5 | P46782 | 23 kDa | 121 | 55 | 4 | 0 |
| Isoform 5 of Tropomyosin alpha-1 chain | P09493 | 28 kDa | 38 | 110 | 4 | 14 |
| Myosin-14 | Q7Z406 | 228 kDa | 21 | 141 | 4 | 27 |
| ATP synthase subunit gamma, mitochondrial | P36542 | 33 kDa | 24 | 2 | 4 | 0 |
| ***MW= Molecular Weight, PQV=Pulldown Quantitative Value, FE=Fold Enrichment, NC = Negative Control Peptide** | | | | | | |

**Supplementary Table 1: Fold enrichment and label free quantification of top proteins in UA-1907/SKMEL-147 pulldown.** Top 40 proteins identified in UA-1907/SKMEL-147 pulldown. Table of proteins from LC-MS/MS gated by label free quantification of >20 and subsequently sorted by fold enrichment. Proteins displayed with description, accession number, and molecular weight. 1907 pulldown results displayed alongside 2014 (inactive peptide control) pulldown results. Fold enrichment values visualized graphically in Figure 2.

| **Biological Processes** | | |
| --- | --- | --- |
| **GO Term** | **Gene Count** | **P-Value** |
| attachment of GPI anchor to protein | 3 | 5.50E-05 |
| intermediate filament organization | 4 | 3.60E-04 |
| keratinization | 4 | 4.20E-04 |
| regulation of focal adhesion assembly | 3 | 1.00E-03 |
| integrin-mediated signaling pathway | 4 | 1.30E-03 |
| mitochondrion organization | 4 | 1.50E-03 |
| GPI anchor biosynthetic process | 3 | 1.90E-03 |
| regulation of cell shape | 4 | 2.40E-03 |
| cellular response to reactive oxygen species | 3 | 3.60E-03 |
| positive regulation of heart rate by epinephrine | 2 | 3.90E-03 |
| substrate adhesion-dependent cell spreading | 3 | 4.00E-03 |
| platelet aggregation | 3 | 4.10E-03 |
| positive regulation of non-canonical NF-kappaB signal transduction | 3 | 7.60E-03 |
| establishment or maintenance of cell polarity regulating cell shape | 2 | 7.80E-03 |
| actin cytoskeleton organization | 4 | 9.40E-03 |
| protein stabilization | 4 | 1.20E-02 |
| positive regulation of GTPase activity | 3 | 1.30E-02 |
| cytoplasmic translation | 3 | 1.40E-02 |
| RIG-I signaling pathway | 2 | 1.40E-02 |
| epidermal growth factor receptor signaling pathway | 3 | 1.70E-02 |
| endothelin receptor signaling pathway | 2 | 1.90E-02 |
| cell adhesion | 5 | 2.30E-02 |
| regulation of muscle contraction | 2 | 2.50E-02 |
| muscle filament sliding | 2 | 3.10E-02 |
| ruffle organization | 2 | 3.10E-02 |
| actin filament organization | 3 | 3.20E-02 |
| cytoskeleton organization | 3 | 3.70E-02 |
| positive regulation of ATP-dependent activity | 2 | 4.80E-02 |
| ventricular cardiac muscle tissue morphogenesis | 2 | 5.00E-02 |

**Supplementary Table 2. Gene Ontology Proteomic Enriched Pathways: Biological Processes.** The table above lists pathway enrichment identified using the NIH DAVID gene ontology tool for biological processes. These tables represent the values used to generate the pie charts in Figure 3. The table is sorted by p-value, a lower p-value correlation to higher pathway enrichment. The table was filtered to have a p-value of 0.05 or lower, identifying only significant pathways.

| **Cellular Component** | | |
| --- | --- | --- |
| **GO Term** | **Gene Count** | **P-Value** |
| focal adhesion | 14 | 8.40E-13 |
| extracellular exosome | 17 | 1.60E-06 |
| cytosol | 25 | 1.60E-05 |
| actin cytoskeleton | 7 | 1.80E-05 |
| GPI-anchor transamidase complex | 3 | 3.60E-05 |
| mitochondrial inner membrane | 7 | 4.00E-04 |
| cytoplasm | 23 | 4.70E-04 |
| stress fiber | 4 | 6.00E-04 |
| keratin filament | 4 | 9.10E-04 |
| membrane | 21 | 9.90E-04 |
| ruffle membrane | 4 | 9.90E-04 |
| small ribosomal subunit | 3 | 1.20E-03 |
| intermediate filament | 4 | 2.30E-03 |
| cytosolic small ribosomal subunit | 3 | 3.10E-03 |
| mitochondrial prohibitin complex | 2 | 3.80E-03 |
| sarcomere | 3 | 4.30E-03 |
| mitochondrion | 10 | 4.60E-03 |
| lamellipodium | 4 | 5.90E-03 |
| muscle thin filament tropomyosin | 2 | 7.70E-03 |
| cytosolic ribosome | 3 | 1.50E-02 |
| ruffle | 3 | 1.60E-02 |
| bleb | 2 | 1.90E-02 |
| cytoskeleton | 5 | 2.70E-02 |
| ribosome | 3 | 4.50E-02 |

|  |
| --- |

**Supplementary Table 3. Gene Ontology Proteomic Enriched Pathways: Cellular Component.** The table above lists pathway enrichment identified using the NIH DAVID gene ontology tool for cellular component. These tables represent the values used to generate the pie charts in Figure 3. The table is sorted by p-value, a lower p-value correlation to higher pathway enrichment. The table was filtered to have a p-value of 0.05 or lower, identifying only significant pathways.

| **Molecular Function** | | |
| --- | --- | --- |
| **GO Term** | **Gene Count** | **P-Value** |
| actin binding | 9 | 5.00E-07 |
| structural constituent of cytoskeleton | 6 | 3.60E-06 |
| structural constituent of skin epidermis | 4 | 6.10E-05 |
| actin filament binding | 6 | 8.30E-05 |
| protein kinase binding | 6 | 3.40E-03 |
| protein binding | 37 | 3.90E-03 |
| integrin binding | 4 | 4.40E-03 |
| cytoskeletal protein binding | 3 | 5.60E-03 |
| protein homodimerization activity | 6 | 1.90E-02 |
| protein heterodimerization activity | 4 | 4.50E-02 |

**Supplementary Table 4. Gene Ontology Proteomic Enriched Pathways: Molecular Function.** The table above lists pathway enrichment identified using the NIH DAVID gene ontology tool for molecular function. These tables represent the values used to generate the pie charts in Figure 3. The table is sorted by p-value, a lower p-value correlation to higher pathway enrichment. The table was filtered to have a p-value of 0.05 or lower, identifying only significant pathways.
