# Supplementary material for: Establishing selectivity of FAK-paxillin PPI inhibitor using pulldown proteomics and a focal adhesion protein selectivity panel
Source: Biochem Biophys Rep. 2025 Dec 14;45:102410. doi: 10.1016/j.bbrep.2025.102410 (PMC12765108; doi:10.1016/j.bbrep.2025.102410)
Supplement: Multimedia component 2 [file mmc2.pdf]

Blot Supplementary Information-

O'Brien, H, et al. Establishing Drug Selectivity of FAK-PPI Inhibitors using Pulldown Proteomics and a Focal Adhesion Protein Selectivity Panel

Table of Contents:

Gel 1. Figure 1B. Triplicate.

Blot 1. Figure 3A FAK Rep. 1

Blot 2. Figure 3A FAK Rep. 2-3

Blot 3. Figure 3A Vinculin Rep. 1

Blot 4. Figure 3A Vinculin Rep. 2-3

Blot 5. Figure 3A Talin-1 Rep. 1

Blot 6. Figure 3A Talin-1 Rep. 2-3

Blot 7. Figure 3A Paxillin Rep. 1

Blot 8. Figure 3A Paxillin Rep. 2-3

Gel 2. Figure 4A FAK-FAT Fractions

Gel 3. Figure 4B Talin-1 Rod 8 Fractions

Gel 4. Figure 4C α-Parvin Fractions

Gel 5. Figure 4D β-Parvin Fractions

Gel 6. Figure 4E Vinculin Head Fractions

Gel 7. Figure 4F Vinculin Tail Fractions

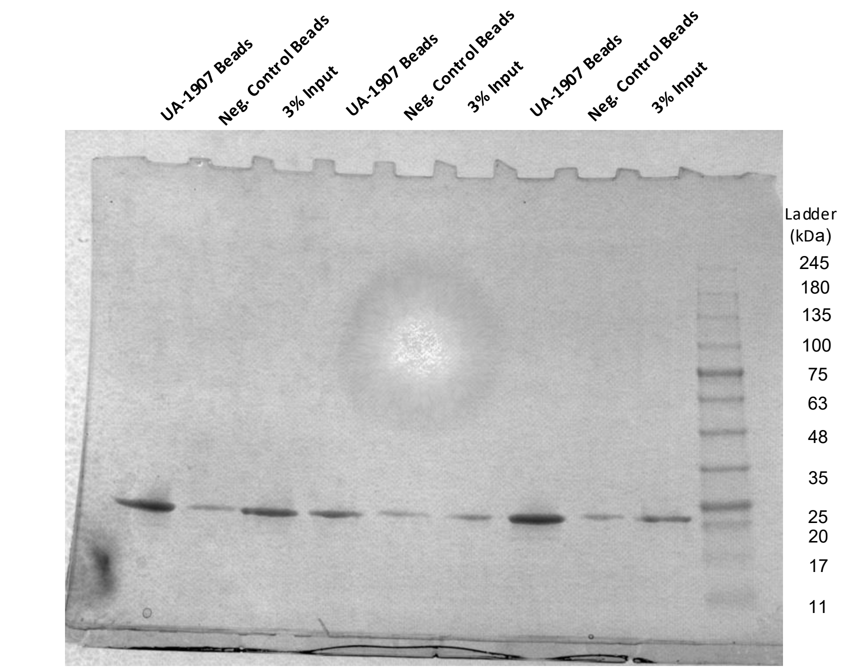

Gel 1. Figure 1B. Triplicate.

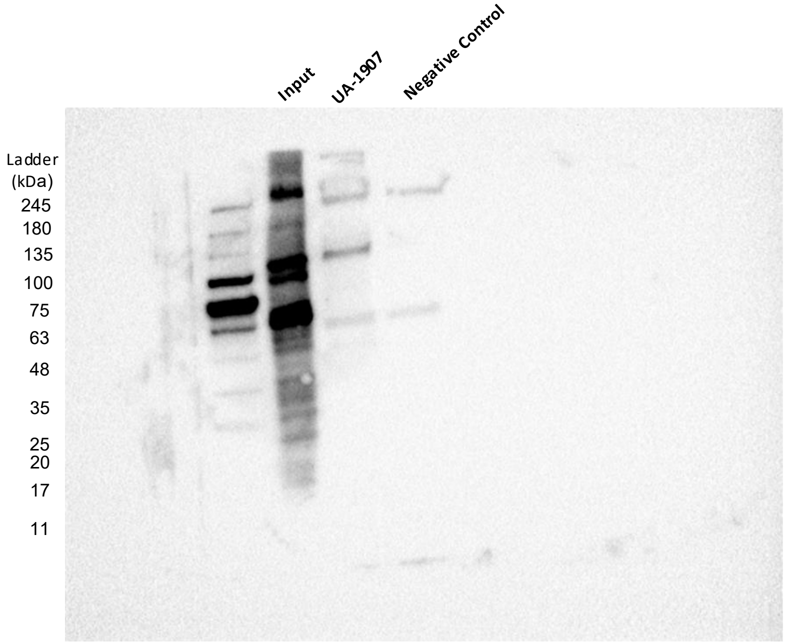

Blot 1. Figure 3A FAK Rep. 1

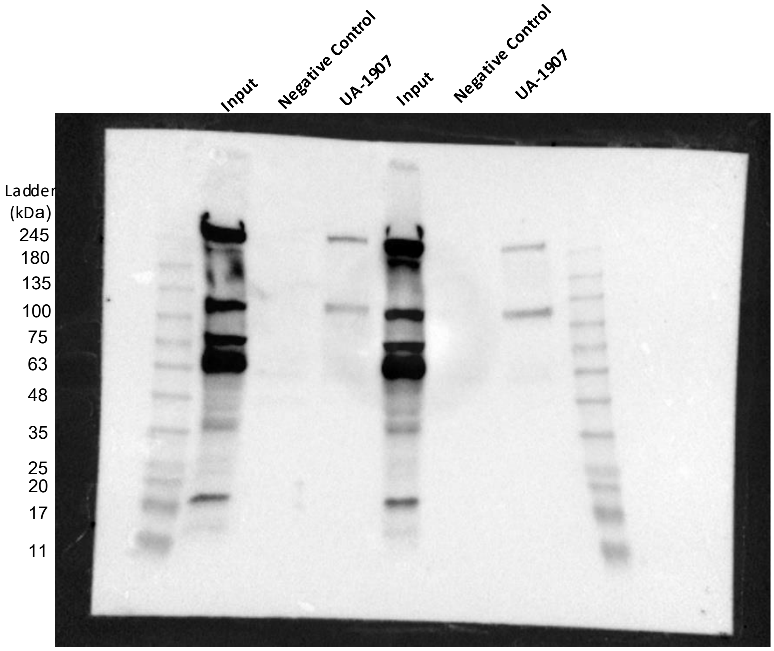

Blot 2. Figure 3A FAK Rep. 2-3

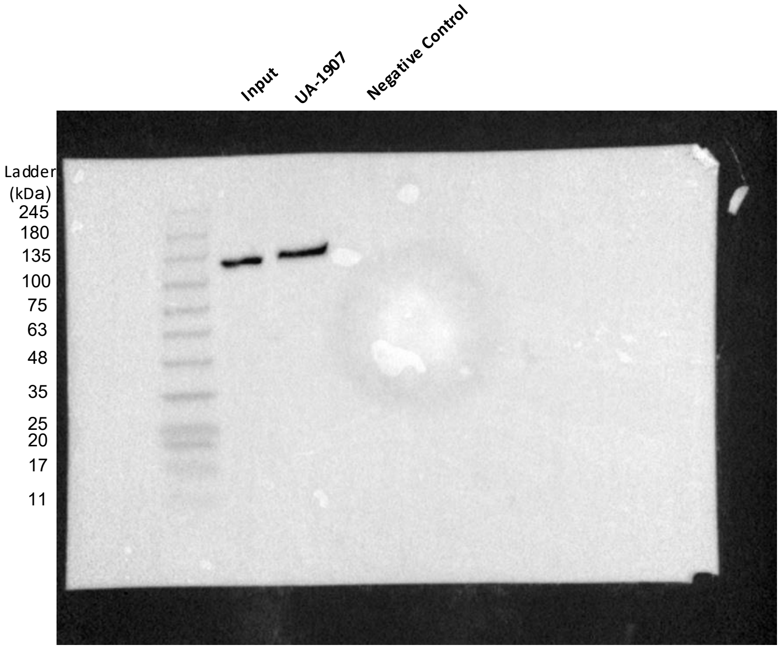

Blot 3. Figure 3A Vinculin Rep. 1

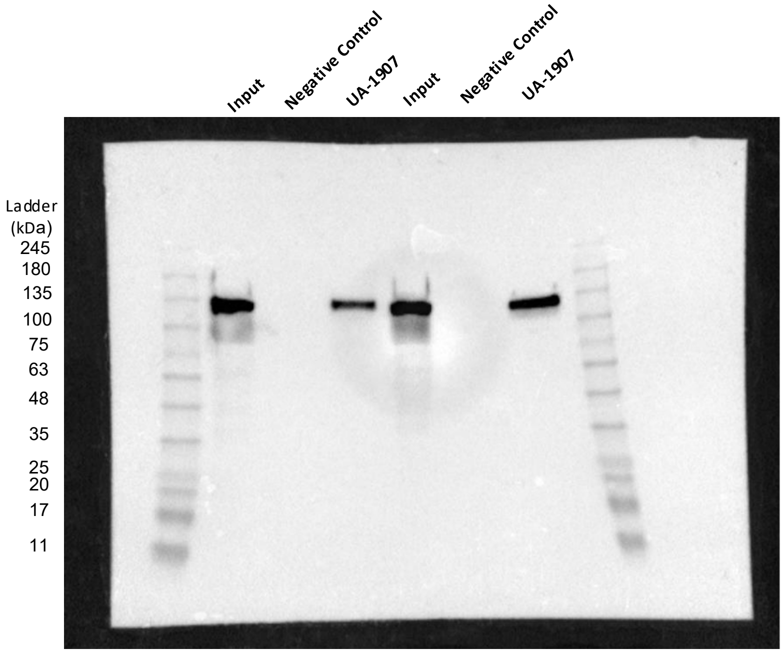

Blot 4. Figure 3A Vinculin Rep. 2-3

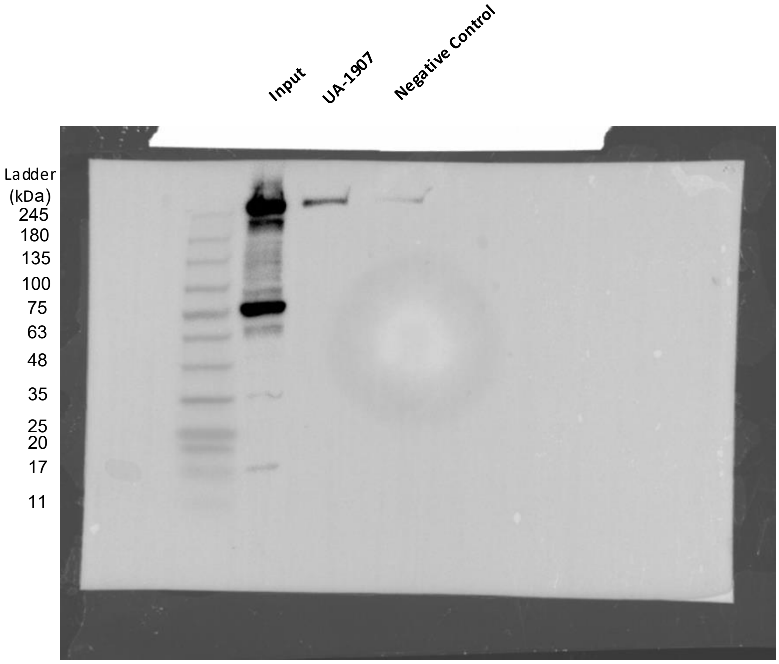

Blot 5. Figure 3A Talin-1 Rep. 1

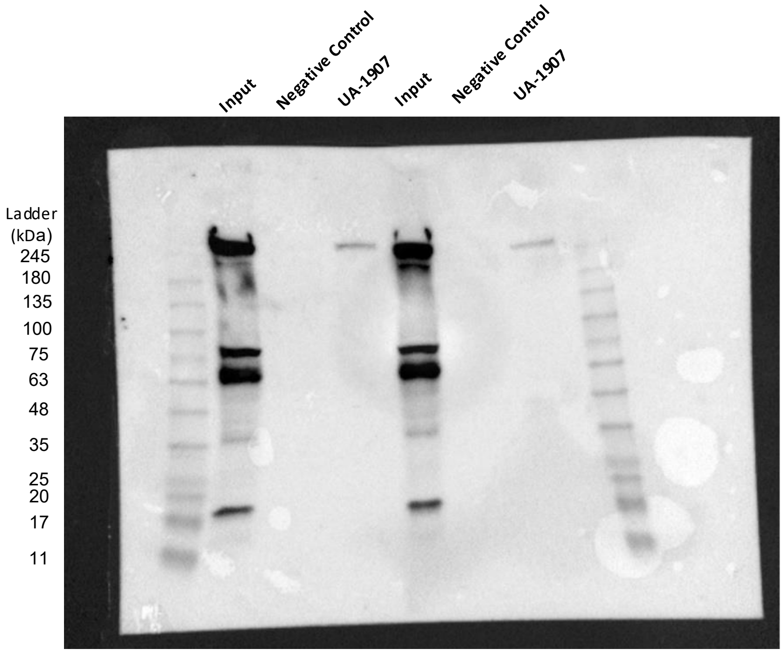

Blot 6. Figure 3A Talin-1 Rep. 2-3

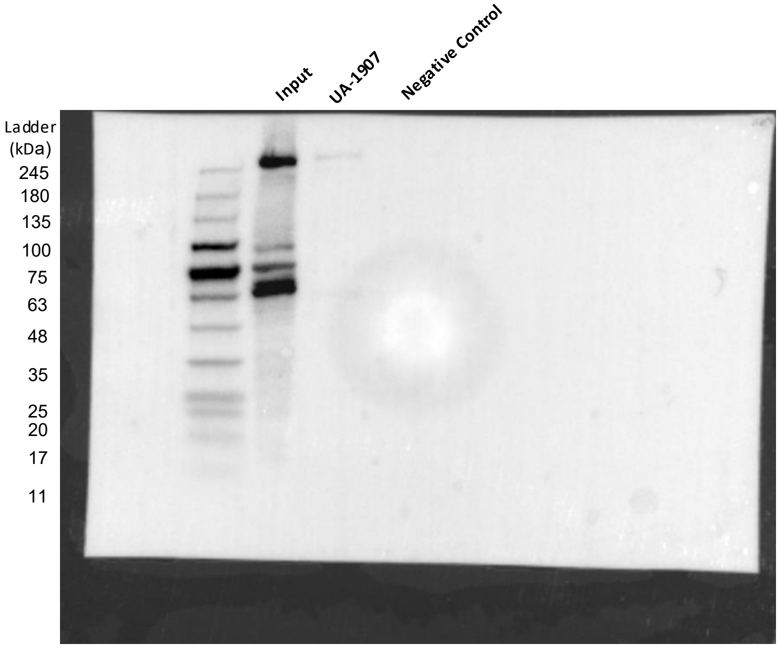

Blot 7. Figure 3A Paxillin Rep. 1

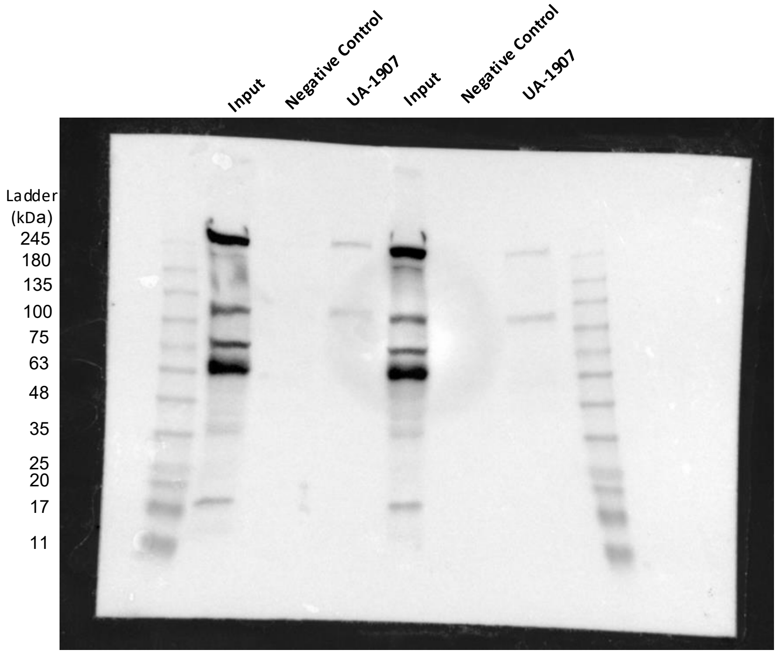

Blot 8. Figure 3A Paxillin Rep. 2-3

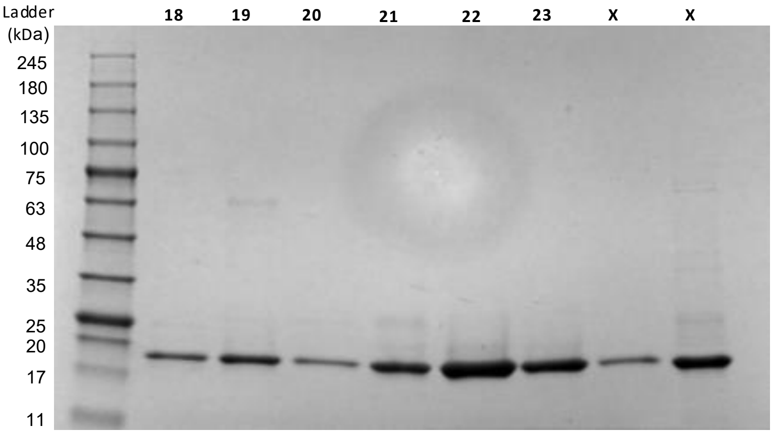

Gel 2. Figure 4A FAK-FAT Fractions

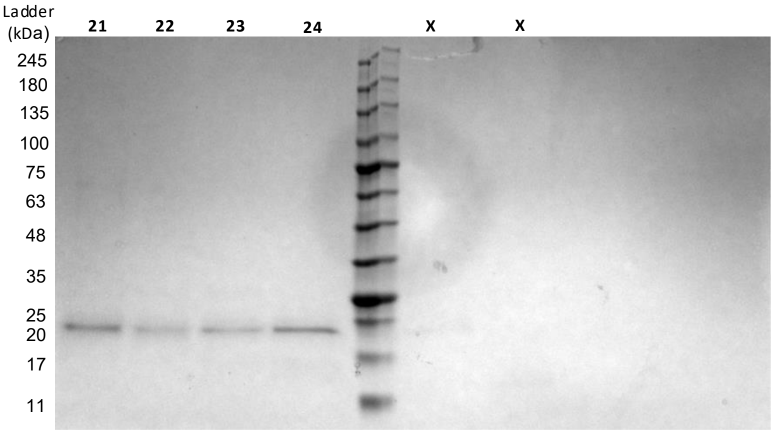

Gel 3. Figure 4B Talin-1 Rod 8 Fractions

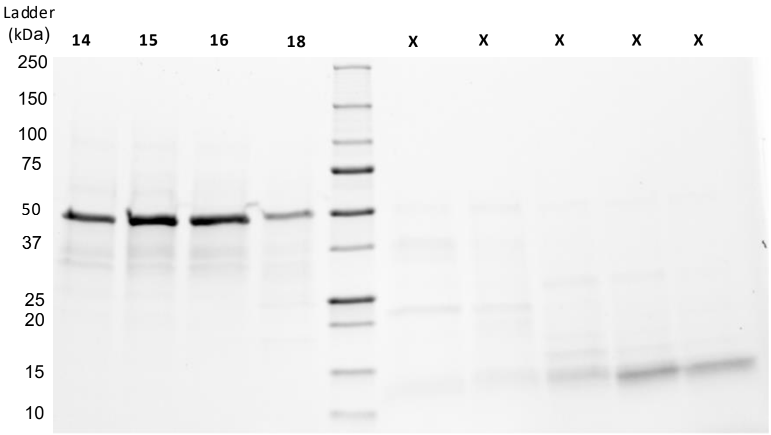

Gel 4. Figure 4C α-Parvin Fractions

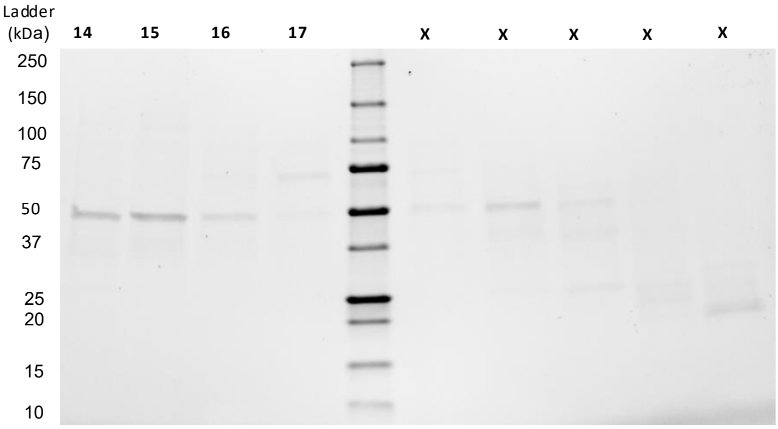

Gel 5. Figure 4D β-Parvin Fractions

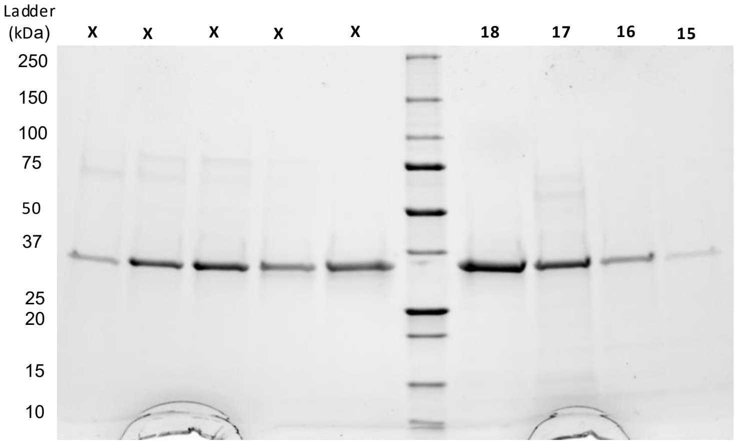

Gel 6. Figure 4E Vinculin Head Fractions

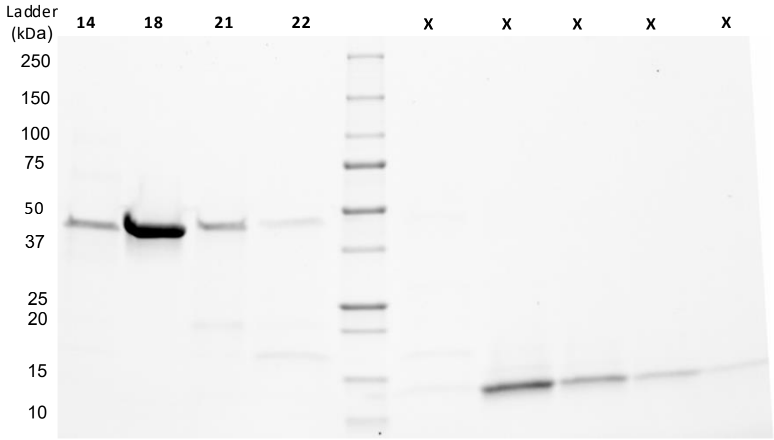

Gel 7. Figure 4F Vinculin Tail Fractions
